# A Convex Optimization Algorithm for Compressed Sensing in a Complex Domain: The Complex-Valued Split Bregman Method

**DOI:** 10.3390/s19204540

**Published:** 2019-10-18

**Authors:** Kai Xiong, Guanghui Zhao, Guangming Shi, Yingbin Wang

**Affiliations:** School of Artificial Intelligence, Xidian University, Xi’an 710071, China; kxiong@stu.xidian.edu.cn (K.X.); gmshi@xidian.edu.cn (G.S.); xidianwangbin@163.com (Y.W.)

**Keywords:** Split Bregman method, Bregman Iteration, complex domain, convex optimization, compressed sensing

## Abstract

The Split Bregman method (SBM), a popular and universal CS reconstruction algorithm for inverse problems with both *l*_1_-norm and TV-norm regularization, has been extensively applied in complex domains through the complex-to-real transforming technique, e.g., MRI imaging and radar. However, SBM still has great potential in complex applications due to the following two points; Bregman Iteration (BI), employed in SBM, may not make good use of the phase information for complex variables. In addition, the converting technique may consume more time. To address that, this paper presents the complex-valued Split Bregman method (CV-SBM), which theoretically generalizes the original SBM into the complex domain. The complex-valued Bregman distance (CV-BD) is first defined by replacing the corresponding regularization in the inverse problem. Then, we propose the complex-valued Bregman Iteration (CV-BI) to solve this new problem. How well-defined and the convergence of CV-BI are analyzed in detail according to the complex-valued calculation rules and optimization theory. These properties prove that CV-BI is able to solve inverse problems if the regularization is convex. Nevertheless, CV-BI needs the help of other algorithms for various kinds of regularization. To avoid the dependence on extra algorithms and simplify the iteration process simultaneously, we adopt the variable separation technique and propose CV-SBM for resolving convex inverse problems. Simulation results on complex-valued *l*_1_-norm problems illustrate the effectiveness of the proposed CV-SBM. CV-SBM exhibits remarkable superiority compared with SBM in the complex-to-real transforming technique. Specifically, in the case of large signal scale *n* = 512, CV-SBM yields 18.2%, 17.6%, and 26.7% lower mean square error (MSE) as well as takes 28.8%, 25.6%, and 23.6% less time cost than the original SBM in 10 dB, 15 dB, and 20 dB SNR situations, respectively.

## 1. Introduction

Compressed sensing (CS) theory has been thoroughly analyzed and extensively applied in the signal processing [1,2] and image processing community [3,4,5] during the past decades. CS theory indicates that a sparse signal can be reconstructed from a few of measurements lower than the Nyquist rate required [6,7]. Specifically, an unknown vector $x\in {\mathbb{R}}^{n}$ can be recovered by solving an inverse problem with a sparsity-promoting regularization term, such as *l*_1_-norm or total variation (TV) norm, as follows:

(1) $$\underset{x}{\min}\frac{\lambda }{2}{\left\|y-Ax\right\|}_{2}^{2}+{\left\|x\right\|}_{1}$$

(2) $$\underset{x}{\min}\frac{\lambda }{2}{\left\|y-Ax\right\|}_{2}^{2}+{\left\|x\right\|}_{TV}$$

where a measurements vector $y\in {\mathbb{R}}^{m}$ is generated by *y* = *Ax* + *ε*, $A\in {\mathbb{R}}^{m\times n}$ with *m* < *n* denotes a sensing matrix, and $\varepsilon \in {\mathbb{R}}^{m}$ represents a noise vector. There are various convex optimization methods [8,9,10,11] and sparse Bayesian learning methods [12,13,14] dealing with related inverse problems. However, the vast majority of the above-mentioned algorithms consider the real-valued situations. In many applications, such as diverse as wireless communication [15,16,17], biomedical [18,19], and radar [20,21,22], the complex domain provides signals and images with a more convenient and appropriate representation to preserve their sparsity and phase information than the real domain. Motived by this, we investigate CS reconstruction methods in complex-valued cases.

In recent years, there have been many papers focusing on this problem, such as Complex Approximate Message Passing (CAMP) [23] and M-lasso [24]. CAMP is the extension of Approximate Message Passing (AMP) [25] to the complex domain. However, the reconstruction performance of CAMP for the unequal-amplitude sparse signal is poor [26]. M-lasso combines the zero subgradient equations with M-estimation settling Equation (1) in the complex domain. However, the updating strategy in M-lasso is cyclic coordinate descent (CCD) [27], which calculates one element at a time while keeping others fixed at the current iteration. Obviously, it is computationally expensive. Furthermore, these schemes are designed for specific *l*_1_-norm regularization problems. Thus, a more general algorithm for CS recovery in complex variables is needed.

The Split Bregman method (SBM) proposed in [28] is a universal convex optimization algorithm for both *l*_1_-norm and TV-norm regularization problems. By the idea of decomposing the original problem into several subproblems worked out by Bregman Iteration (BI) [29,30], SBM has been widely utilized in the complex domain through the complex-to-real converting technique [31,32], e.g., MRI imaging [33], SAR imaging [34], forward-looking scanning radar imaging [35], SAR image super-resolution [36], and massive MIMO channel estimation [37]. However, SBM still has great potential in terms of both reconstruction performance and time cost considering the following two points: The original BI defined in the real domain may not make good use of the phase information for complex variables, which degrades the recovery accuracy; secondly, the converting technique quadruples the elements of the sensing matrix *A* to 2*m* × 2*n*, which consumes more memory and time within the iteration process.

To tackle the aforementioned problems, this paper theoretically generalizes the original SBM into the complex domain, named the complex-valued Split Bregman method (CV-SBM). We first define the complex-valued Bregman distance (CV-BD) and replace the associated regularization term with the CV-BD in the inverse problem. Then, complex-valued Bregman Iteration (CV-BI) is proposed to solve this new problem. In addition, according to the calculation rules, Wirtinger’s Calculus, and optimization theory for complex variables, how well-defined the CV-BI is and its convergence are analyzed in detail. The proof of the above two properties reveals that CV-BI can settle inverse problems if the regularization term is convex. Since CV-BI requires the help of additional algorithms to find the solution to the specific regularization, as BI does, its solution is still complicated and computationally expensive. Inspired by SBM, we adopt the variable separation technique to avoid the requirement of other optimization algorithms and then present CV-SBM to settle the convex inverse problems with the simplified solution. Simulation results on the complex-valued *l*_1_-norm problems reveal the effectiveness of CV-SBM compared with existing methods. Particularly, the proposed CV-SBM exhibits 18.2%, 17.6%, and 26.7% lower mean square error (MSE) and takes 28.8%, 25.6%, and 23.6% less time than SBM through the complex-to-real transforming technique in 10 dB, 15 dB and 20 dB SNR cases with large signal scale *n* = 512.

The rest of this paper is organized as follows. In Section 2, we briefly review the original BI and SBM techniques. Section 3 proposes and analyzes CV-BI and CV-SBM in detail. Section 4 conducts numerical experiments and compares the results with some existing CS reconstruction algorithms in the complex domain. Conclusions and future work are discussed in Section 5.

## 2. Review of Bregman Iteration and Split Bregman Method

SBM, whose main idea is to decompose the original unconstrained problem into several equivalent subproblems solved by BI [29,30], has shown its efficiency and effectiveness for inverse problems with both *l*_1_-norm and TV-norm regularization [28]. For the convenience of the following illustration, we will first present a brief review of BI and SBM.

### 2.1. Bregman Iteration

BI focuses on the optimization problem

(3) $$\underset{x\in \mathbb{R}}{\min}J\left(x\right)+H\left(x\right)$$

where *J*(*x*) is a real convex function but not necessarily differentiable, and *H* is a real convex and differentiable function. By replacing *J*(*x*) with corresponding Bregman distance (BD) $D_{J}^{p}\left(u,v\right)$ [29,38], BI tackles Equation (3) as follows:

(4) $$D_{J}^{p}\left(u,v\right)=J\left(u\right)-J\left(v\right)-\langle p,u-v\rangle$$

(5) $$x^{k}=\arg\underset{x}{\min}D_{J}^{p_{k-1}}\left(x,x^{k-1}\right)+H\left(x\right)$$

(6) $$p^{k}=p^{k-1}-\nabla H\left(x^{k}\right)$$

where p∈∂J(v) is a subgradient of *J*(*x*) at the point *v*, and ∂J(v) is the subdifferential of *J*(*x*) at *v*, $\langle f,g\rangle =f^{T}g^{*}$ denotes the inner product for all $f,g\in {\mathbb{C}}^{n}$, and (·)* is used for the conjugate. To make it clear, we give the definition of the subgradient and subdifferential.

**Definition** **1.** Let $T:{\mathbb{R}}^{m}\to \mathbb{R}$ be a convex function defined on the real domain. A vector $\nabla_{x}^{s}T\left(z_{0}\right)\in {\mathbb{R}}^{m}$ is said to be a subgradient of *T* at *z*_0_ if $T\left(z\right)\geq T\left(z_{0}\right)+\langle z-z_{0},\nabla_{x}^{s}T\left(z_{0}\right)\rangle$. The set of all subgradients of *T* at *z*_0_ is called the subdifferential of *T* at *z*_0_ and is denoted by $\partial_{z}T\left(z_{0}\right)$. More detials about the subgradient and subdifferential can be found in [39].

A key property of the BD is that it has the same convex characteristic as *J*(*x*) so that (5) is still a convex problem. Furthermore, it can be used as the measurement of the closeness of two points in *J*(*x*) [29,30].

As for the *l*_1_-norm and TV-norm problems, Equation (6) is easily calculated, whereas dealing with (5) is more complicated and needs the help of another algorithm [30], such as GPSR [8] or FPC [9]. To avoid the dependence on extra algorithms and simplify the iteration process simultaneously, SBM is presented.

### 2.2. Split Bregman Method

SBM aims to find a solution to the unconstrained problem

(7) $$\underset{x}{\min}{\left\|\Phi x\right\|}_{1}+H\left(x\right)$$

where Φ is a linear operator. SBM introduces an auxiliary variable *d* and considers an equivalent constrained problem to Equation (7)

(8) $$\underset{x,d}{\min}{\left\|d\right\|}_{1}+H\left(x\right)\;s.t.\;d=\Phi x$$

The corresponding unconstrained version of (8) can be formulated as follows:

(9) $$\underset{x,d}{\min}E\left(x,d\right)+\frac{\mu }{2}{\left\|d-\Phi x\right\|}_{2}^{2}$$

where *E*(*x*,*d*) = ||*d*||_1_ + *H*(*x*) and *μ* is a positive constant to balance the two terms in Equation (9) within the iterations. Since there are two unknown variables completely fulfilling the demand of BI for optimization problems, BI can be performed for each of them:

(10) $$\begin{array}{ll} \left(x^{k},d^{k}\right) & =\arg\underset{x,d}{\min}D_{E}^{p_{k-1}}\left(x,x^{k-1},d,d^{k-1}\right)+\frac{\mu }{2}{\left\|d-\Phi x\right\|}_{2}^{2}\\ & =\arg\underset{x,d}{\min}E\left(x,d\right)-E\left(x^{k-1},d^{k-1}\right)-\langle p_{x}^{k-1},x-x^{k-1}\rangle -\langle p_{d}^{k-1},d-d^{k-1}\rangle +\frac{\mu }{2}{\left\|d-\Phi x\right\|}_{2}^{2} \end{array}$$

(11) $$p_{x}^{k}=p_{x}^{k-1}-\mu \Phi^{T}\left(\Phi x^{k}-d^{k}\right)$$

(12) $$p_{d}^{k}=p_{d}^{k-1}-\mu \left(d^{k}-\Phi x^{k}\right)$$

Let $b^{k}=p_{d}^{k}/\mu$, then the iterations become:

(13) $$\left(x^{k},d^{k}\right)=\arg\underset{x,d}{\min}{\left\|d\right\|}_{1}+H\left(x\right)+\frac{\mu }{2}{\left\|d-\Phi x-b^{k-1}\right\|}_{2}^{2}$$

(14) $$b^{k}=b^{k-1}-\left(d^{k}-\Phi x^{k}\right)$$

For (13), one can decompose it into resolving two subproblems alternately, i.e., working out *x_k_* by fixing *d_k_*_−1_ and then clearing up *d_k_* by fixing *x_k_*

(15) $$x^{k}=\arg\underset{x}{\min}H\left(x\right)+\frac{\mu }{2}{\left\|d^{k-1}-\Phi x-b^{k-1}\right\|}_{2}^{2}$$

(16) $$d^{k}=\arg\underset{d}{\min}{\left\|d\right\|}_{1}+\frac{\mu }{2}{\left\|d-\Phi x^{k}-b^{k-1}\right\|}_{2}^{2}$$

The problems above can be computed conveniently by tackling zero subgradient equations.

Evidently, the combination of BI and SBM can be adopted to settle plenty of convex optimization problems in a real system [40,41]. However, BD and BI are established in the real domain, and consequently do not to take complex variables and phase information into account. Specifically, once variables come in the complex domain, the BD becomes complex-valued and consequently cannot be employed as the measurement of the closeness. Thus, we can no longer use the BD as the objective function. In the following section, we will generalize the original BI and SBM into the complex domain theoretically.

## 3. Complex-Valued Split Bregman Method

### 3.1. Wirtinger Calculus and Wirtinger’s Subgradients

As is well-known, convex optimization theory requires the differentiability of the objective function. For *T*(*c*) = *T_R_*(*c*) + *jT_I_*(*c*) in complex variables *c* = *c_R_* + *jc_I_*, the complex differentiability equals to the satisfied Cauchy–Riemann conditions:

(17) $$\begin{array}{l} \frac{\partial T_{R}\left(c\right)}{\partial c_{R}}=\frac{\partial T_{I}\left(c\right)}{\partial c_{I}}\\ \frac{\partial T_{R}\left(c\right)}{\partial c_{I}}=-\frac{\partial T_{I}\left(c\right)}{\partial c_{R}} \end{array}$$

For a complex-valued *l*_1_-norm regularization problem:

(18) $$\begin{array}{c} \underset{x\in {\mathbb{C}}^{n}}{\min}F\left(x\right)\\ F\left(x\right)=H\left(x\right)+J\left(x\right) \end{array}$$

where $H\left(x\right)=\lambda {\left\|y-Ax\right\|}_{2}^{2}$, $J\left(x\right)={\left\|x\right\|}_{1}$, $y\in {\mathbb{C}}^{m}$, $A\in {\mathbb{C}}^{m\times n}$. Apparently, *F*(*x*) does not obey (17) so that calculating the complex gradient directly is unavailable. 

To overcome such a problem, an alternative tool for computing the complex gradient was brought into light recently called Wirtinger’s calculus [39]. It relaxes the strict requirement for complex differentiability and allows the computation of the complex gradient in simple rules and principles. A key point in Wirtinger’s calculus is the definition of Wirtinger’s gradient (W-gradient) and the conjugate Wirtinger’s gradient (CW-gradient)

(19) $$\nabla_{c}T\left(c\right)=\frac{1}{2}\left(\nabla_{c_{R}}T\left(c\right)-j\nabla_{c_{I}}T\left(c\right)\right)$$

(20) $$\nabla_{c*}T\left(c\right)=\frac{1}{2}\left(\nabla_{c_{R}}T\left(c\right)+j\nabla_{c_{I}}T\left(c\right)\right)$$

where $\nabla_{c_{R}}T\left(c\right)$ and $\nabla_{c_{I}}T\left(c\right)$ represent the gradient of the *T* at *c_R_* and *c_I_*, which can be obtained by the traditional ways. According to Equations (19) and (20), one can calculate the W-gradient of c^*^ and the CW-gradient of *c*:

(21) $$\begin{array}{ll} \nabla_{c}c^{*} & =\frac{1}{2}\left(\nabla_{c_{R}}c^{*}-j\nabla_{c_{I}}c^{*}\right)=\frac{1}{2}\left[\nabla_{c_{R}}\left(c_{R}-jc_{I}\right)-j\nabla_{c_{I}}\left(c_{R}-jc_{I}\right)\right]\\ & =\frac{1}{2}\left[1-j\left(-j\right)\right]=\frac{1}{2}\left[1-1\right]=0 \end{array}$$

(22) $$\begin{array}{ll} \nabla_{c^{*}}c & =\frac{1}{2}\left(\nabla_{c_{R}}c+j\nabla_{c_{I}}c\right)=\frac{1}{2}\left[\nabla_{c_{R}}\left(c_{R}+jc_{I}\right)+j\nabla_{c_{I}}\left(c_{R}+jc_{I}\right)\right]\\ & =\frac{1}{2}\left[1+j\left(j\right)\right]=\frac{1}{2}\left[1-1\right]=0 \end{array}$$

Considering that both the W-gradient for c^*^ and the CW-gradient for *c* are equal to zero, in Wirtinger’s calculus we can treat *c* and c^*^ as two irrelevant or independent variables, which is the main approach allowing us to utilize the elegance of Wirtinger’s calculus. Here is an example: if *T*(*c*) = *c*(c^*^)^2^, then we have $\nabla_{c}T\left(c\right)={\left(c^{*}\right)}^{2}$ and $\nabla_{c^{*}}T\left(c\right)=2cc^{*}$. More details and examples can be found in [42].

In general, for the convex function in complex variables, the optimization condition is the CW-gradient equal to zeros vector. Nevertheless, in practice, some functions may not be differentiable everywhere, e.g., *l*_1_-norm in *F*(*x*) at zero. In this case, the conjugate Wirtinger’s subgradients (CW-subgradients) [39] can be adopted to construct the gradient path towards the optimal point. For a real convex function in complex variables $T:{\mathbb{C}}^{n}\to \mathbb{R}$, we define a CW-subgradient $\nabla_{c^{*}}^{s}T\left(c\right)$ of *T* at *c* if $\forall c_{0}\in {\mathbb{C}}^{n}$

(23) $$\nabla_{c^{*}}^{s}T\left(c\right)=1/2\left[\nabla_{c_{R}}^{s}T\left(c\right)+j\nabla_{c_{I}}^{s}T\left(c\right)\right]$$

and it satisfies

(24) $$T\left(c+c_{0}\right)\geq T\left(c\right)+2ℜ\left(\langle c,\nabla_{c^{*}}^{s}T\left(c\right)\rangle \right)=T\left(c\right)+\langle c_{R},\nabla_{c_{R}}^{s}T\left(c\right)\rangle +\langle c_{I},\nabla_{c_{I}}^{s}T\left(c\right)\rangle$$

where $\nabla_{c_{R}}^{s}T\left(c\right)$ and $\nabla_{c_{I}}^{s}T\left(c\right)$ denote the subgradient of *T* at *c_R_* and *c_I_*. The set of all CW-subgradients of *T* at *c* is called Wirtinger’s differential of *T* at *c* and is represented by $\partial_{c^{*}}T\left(c\right)$. It should be noted that for the differentiable point of *T*, the Wirtinger’s differential only contains one element, i.e., its CW-gradient. Wirtinger’s differential of modulus |*x_i_*| and H(x) are presented as follows [43].

(25) $$\partial_{x^{*}}\left|x_{i}\right|=\left\{ \begin{array}{l} \frac{1}{2}\mathrm{sign}\left(x_{i}\right),\;\mathrm{for}\;x_{i}\neq 0\\ \frac{1}{2}s\;\mathrm{for}\;x_{i}=0 \end{array}\right.$$

(26) $$\partial_{x^{*}}H\left(x\right)=\nabla_{x^{*}}H\left(x\right)=\lambda A^{H}\left(Ax-y\right)$$

where *i* is the index for the element of vector *x* and *s* is some complex number verifying |s|≤1. Then, a necessary and sufficient condition for the optimization solution to Equation (18) is that $0\in \partial_{x^{*}}F\left(x\right)$ [43]. By the definition of the CW-subgradient, in the following subsection, we can generalize BD into the complex domain.

### 3.2. CV Bregman Distance

To prevent the BD becoming complex-valued, we first generalize the BD into the complex domain and introduce the CV Bregman distance (CV-BD) theoretically.

**Definition** **2.** For $p=\nabla_{v^{*}}^{s}T\left(v\right)\in \partial_{v^{*}}T\left(v\right)$, we define the quantity

(27) $$\begin{array}{ll} D_{J}^{p}\left(u,v\right) & =J\left(u\right)-J\left(v\right)-2ℜ\left(\langle u-v,p\rangle \right)\\ & =J\left(u\right)-J\left(v\right)-\langle u_{R}-v_{R},\nabla_{u_{R}}^{s}T\left(c\right)\rangle -\langle u_{I}-v_{I},\nabla_{u_{I}}^{s}T\left(c\right)\rangle \end{array}$$

as a CV-BD associated with real convex function J in complex variables. Clearly, no matter whether the variables u and v are in the real or the complex domain, $D_{J}^{p}\left(u,v\right)$ is always a real-valued scalar. According to (24), one can point out that a CV-BD is non-negative.

To ensure that the CV-BD can be utilized as the objective function as the BD, in the following **Lemma 1** and **Lemma 2** prove that the CV-BD is as the same convex as *J*(*x*) and can measure the closeness at two points in *J*.

**Lemma** **1.** *Let*$D_{J}^{p}\left(u,v\right)$*be a CV-BD associated with real convex or strictly convex function J, where*$u,v\in {\mathbb{C}}^{n}$. *Then*$D_{J}^{p}\left(u,v\right)$*is as the same convex property as J for variable u in each v.*

**Proof.**  Assume *J* is a real convex function and let ∀θ∈[0,1], $\forall x,y,v\in {\mathbb{C}}^{n}$, and $p\in \partial_{v^{*}}J\left(v\right)$. Then we get

(28) $$\begin{array}{ll} D_{J}^{p}\left(\theta x+\left(1-\theta \right)y,v\right) & =J\left(\theta x+\left(1-\theta \right)y\right)-J\left(v\right)-2ℜ\left(\langle \theta x+\left(1-\theta \right)y-v,p\rangle \right)\\ & =J\left(\theta x+\left(1-\theta \right)y\right)-J\left(v\right)+C_{0} \end{array}$$

(29) $$\begin{array}{ll} \theta D_{J}^{p}\left(x,v\right)+\left(1-\theta \right)D_{J}^{p}\left(y,v\right) & =\theta \left[J\left(x\right)-J\left(v\right)-2ℜ\left(\langle x-v,p\rangle \right)\right]\\ & \;+\left(1-\theta \right)\left[J\left(y\right)-J\left(v\right)-2ℜ\left(\langle y-v,p\rangle \right)\right]\\ & =\theta J\left(x\right)+\left(1-\theta \right)J\left(y\right)-J\left(v\right)-2ℜ\left(\langle \theta x+\left(1-\theta \right)y-v,p\rangle \right)\\ & =\theta J\left(x\right)+\left(1-\theta \right)J\left(y\right)-J\left(v\right)+C_{0} \end{array}$$

where $C_{0}=-2ℜ\left(\langle \theta x+\left(1-\theta \right)y-v,p\rangle \right)$.

Considering that *J* is a real convex function, *J* satisfies

(30) J(θx+(1−θ)y)≤θJ(x)+(1−θ)J(y)

Then we have

(31) $$D_{J}^{p}\left(\theta x+\left(1-\theta \right)y,v\right)\leq \theta D_{J}^{p}\left(x,v\right)+\left(1-\theta \right)D_{J}^{p}\left(y,v\right)$$

This completes the proof of $D_{J}^{p}\left(u,v\right)$ is a convex function for variable *u* as *J*.

For *J* is a real strictly convex function, we assume ∀θ∈(0,1), $\forall x,y,v\in {\mathbb{C}}^{n}$ and x≠y. And *J* satisfies

(32) J(θx+(1−θ)y)<θJ(x)+(1−θ)J(y)

Then according to Equations (28), (29), and (32) we obtain

(33) $$D_{J}^{p}\left(\theta x+\left(1-\theta \right)y,v\right)<\theta D_{J}^{p}\left(x,v\right)+\left(1-\theta \right)D_{J}^{p}\left(y,v\right)$$

which proves that $D_{J}^{p}\left(u,v\right)$ is a strictly convex function for variable *u* as *J*. Then, it can be concluded that $D_{J}^{p}\left(u,v\right)$ is as the same convex property as *J* for variable *u* in each *v*. □

**Lemma 2.**  *Let* $D_{J}^{p}\left(u,v\right)$ *be a CV-BD associated with real strictly convex function J and assume a point* w=θu+(1−θ)v *is on the line segment connecting u and v, where* $u,v\in {\mathbb{C}}^{n}$ *,* θ∈(0,1) *. Then* $D_{J}^{p}\left(u,v\right)\geq D_{J}^{p}\left(w,v\right)$ *and equality holds if and only if u = v.*

**Proof.**  Assume u≠v, then we derive according to **Lemma 1**

(34) $$\begin{array}{ll} D_{J}^{p}\left(w,v\right)=D_{J}^{p}\left(\theta u+\left(1-\theta \right)v,v\right) & <\theta D_{J}^{p}\left(u,v\right)+\left(1-\theta \right)D_{J}^{p}\left(v,v\right)\\ & =\theta D_{J}^{p}\left(u,v\right)<D_{J}^{p}\left(u,v\right) \end{array}$$

when u=v, we yield

(35) $$D_{J}^{p}\left(w,v\right)=D_{J}^{p}\left(\theta u+\left(1-\theta \right)v,v\right)=D_{J}^{p}\left(v,v\right)=D_{J}^{p}\left(u,v\right)=0$$

This completes the proof of **Lemma 2**. Then, the CV-BD at two points in convex function *J* would decrease when they get closer, and may become zero if and only if the two points coincide. This property makes the CV-BD the measurement of closeness at two points.

Thus, inspired by the original BI, we use the CV-BD between the variables to be solved and the current solution to replace real convex function *J*(*x*) as the objective function:

(36) $$\begin{array}{c} x_{k}=\arg\underset{x\in {\mathbb{C}}^{n}}{\min}Q_{k}\left(x\right)\\ Q_{k}\left(x\right)=H\left(x\right)+D_{J}^{p_{k-1}}\left(x,x_{k-1}\right) \end{array}$$

Within the iterations, the CV-BD is nonincreasing. This will be proved in the next subsection. □

Obviously, $Q_{k}\left(x\right)$ is convex because of *H*(*x*) and the CV-BD. However, the CV-BD $D_{J}^{p_{k-1}}\left(x,x_{k-1}\right)$ may be multivalued at nondifferential *x_k_*_-1_, which inevitably interferes with the solution of *x_k_*. As we shall prove below, this issue is not vital, since CV-BI introduced in the following subsection automatically chooses a suitable CW-subgradient when dealing with Equation (36).

### 3.3. CV Bregman Iterations

CV-BI for Equation (36) is proposed directly and the definition and the convergence are proved in the following.

#### 3.3.1. CV-BI Algorithm

Algorithm 1. Let *x*_0_ = 0, *p*_0_ = 0, for *k* = 1,2,

1. compute *x_k_* as a minimizer of the convex function *Q_k_*(*x*)

(37) $$\begin{array}{c} x_{k}=\arg\underset{x\in {\mathbb{C}}^{n}}{\min}Q_{k}\left(x\right)\\ Q_{k}\left(x\right)=\lambda {\left\|y-Ax\right\|}_{2}^{2}+J\left(x\right)-J\left(x_{k-1}\right)-2ℜ\left(\langle x-x_{k-1},p_{k-1}\rangle \right) \end{array}$$

2. compute $p_{k}=p_{k-1}-\lambda A^{H}\left(Ax-y\right)\in \partial_{x^{*}}J\left(x_{k}\right)$

Generally, we can initialize *x*_0_ and *p*_0_ whatever satisfy $p_{0}\in \partial_{x^{*}}J\left(x_{0}\right)$. Nevertheless, for any *x*_0_≠0, its CW-subgradient requires optional calculation, which is not desired in practical.

#### 3.3.2. Definition of the Iteration

In this subsection, we reveal that the iterative procedure in Algorithm 1 is well defined. Specifically, a minimizer *x_k_* exists in *Q_k_*(*x*) and the iteration can find an appropriate CW-subgradient *p_k_* automatically.

**Proposition** **1.**  *Assume that*$H\left(x\right)=\lambda {\left\|y-Ax\right\|}_{2}^{2}$, *J(x) is convex and bounded, and let x_0_=0,*$p_{0}=0\in \partial_{x^{*}}J\left(x_{0}\right)$*. Then, for each*k∈ℕ*, there exists a minimizer x_k_ in Q_k_(x), and there exists an appropriate CW-subgradient*$p_{k}\in \partial_{x^{*}}J\left(x_{k}\right)$*and*$q_{k}=\partial_{x^{*}}H\left(x_{k}\right)=\lambda A^{H}\left(Ax_{k}-y\right)$*such that*

(38) $$p_{k}+q_{k}=p_{k-1}$$

*Moreover, if A has no null space, a minimizer x_k_ is unique.*

**Proof.**  We prove the result by induction. In the case of *k* = 1, *Q*_1_(*x*) becomes the original function *F*(*x*), of which the existence of minimizers and the optimality condition *p*_1_ + *q*_1_ = *p*_0_ = 0 is well known [44]. In addition, assume $r_{k}=\lambda \left(y-Ax_{k}\right)$ and we have *p*_1_ = *A^H^r*_1_.

Then, we proceed from *k*−1 to *k* and assume $p_{k-1}=A^{H}r_{k-1}$ exists. To prove that the minimizers exist, we first discus the boundedness of *Q_k_*(*x*). Recalling the *l*_2_-norm greater than or equal to zero, *Q_k_*(*x*) can be estimated as

(39) $$\begin{array}{ll} Q_{k}\left(x\right) & =J\left(x\right)-J\left(x_{k-1}\right)-2ℜ\left(\langle x-x_{k-1},p_{k-1}\rangle \right)+\lambda {\left\|y-Ax\right\|}_{2}^{2}\\ & =J\left(x\right)-J\left(x_{k-1}\right)-2ℜ\left(\langle x-x_{k-1},A^{H}r_{k-1}\rangle \right)+\lambda {\left\|y-Ax\right\|}_{2}^{2}\\ & =J\left(x\right)-J\left(x_{k-1}\right)-2ℜ\left(\langle Ax-Ax_{k-1},r_{k-1}\rangle \right)+\lambda {\left\|y-Ax\right\|}_{2}^{2}\\ & =J\left(x\right)-J\left(x_{k-1}\right)-2ℜ\left(\langle y-Ax_{k-1},r_{k-1}\rangle -\langle y-Ax,r_{k-1}\rangle \right)+\lambda {\left\|y-Ax\right\|}_{2}^{2}\\ & =J\left(x\right)-J\left(x_{k-1}\right)-2ℜ\left(\frac{1}{\lambda }\langle r_{k-1},r_{k-1}\rangle \right)+\langle y-Ax,r_{k-1}\rangle +{\left(\langle y-Ax,r_{k-1}\rangle \right)}^{*}+\lambda {\left\|y-Ax\right\|}_{2}^{2}\\ & =J\left(x\right)-J\left(x_{k-1}\right)-\frac{2{\left\|r_{k-1}\right\|}_{2}^{2}}{\lambda }+\lambda {\left\|y-Ax+\frac{r_{k-1}}{\lambda }\right\|}_{2}^{2}-\frac{{\left\|r_{k-1}\right\|}_{2}^{2}}{\lambda }\\ & =J\left(x\right)-J\left(x_{k-1}\right)-\frac{3{\left\|r_{k-1}\right\|}_{2}^{2}}{\lambda }+\lambda {\left\|y-Ax+\frac{r_{k-1}}{\lambda }\right\|}_{2}^{2}\\ & \geq J\left(x\right)-J\left(x_{k-1}\right)-\frac{3{\left\|r_{k-1}\right\|}_{2}^{2}}{\lambda } \end{array}$$

Since only *J*(*x*) is not constant, the boundedness of *Q_k_*(*x*) implies the boundedness of *J(x*). This shows that the level sets of *Q_k_* are weak-* compact [29]. Hence, a minimizer of *Q_k_* exists due to the optimization theory. Besides, if *A* has no null space and *H*(*x*) as well as *J*(*x*) is strictly convex, *Q_k_*(*x*) is also strictly convex, and therefore the minimizer is unique. This completes the proof of the existence of minimizers for all *k* > 1.

The following proves *p_k_* and *q_k_* exist for all *k* > 1. According to the optimality conditions for *Q_k_*(*x*)

(40) $$\begin{array}{ll} 0\in \partial_{x^{*}}Q_{k}\left(x\right) & =\partial_{x^{*}}H\left(x_{k}\right)+\partial_{x^{*}}J\left(x_{k}\right)-\partial_{x^{*}}2ℜ\left(\langle x-x_{k-1},p_{k-1}\rangle \right)\\ & =\partial_{x^{*}}H\left(x_{k}\right)+\partial_{x^{*}}J\left(x_{k}\right)-\partial_{x^{*}}\langle x-x_{k-1},p_{k-1}\rangle -\partial_{x^{*}}{\left(\langle x-x_{k-1},p_{k-1}\rangle \right)}^{*}\\ & =\partial_{x^{*}}H\left(x_{k}\right)+\partial_{x^{*}}J\left(x_{k}\right)-p_{k-1} \end{array}$$

we derive that

(41) $$p_{k-1}\in \partial_{x^{*}}H\left(x_{k}\right)+\partial_{x^{*}}J\left(x_{k}\right)$$

Recalling that assume *p_k_*_−1_ exists, one can get that $\partial_{x^{*}}J\left(x_{k}\right)$ and $\partial_{x^{*}}H\left(x_{k}\right)$ are not null sets, and consequently yields the existence of $p_{k}\in \partial_{x^{*}}J\left(x_{k}\right)$ and $q_{k}=\partial_{x^{*}}H\left(x_{k}\right)=\lambda A^{H}\left(Ax-y\right)$, which also satisfies Equation (38).

Recalling Equation (38) and *p*_0_ = 0, we obtain that

(42) $$p_{k}=-\sum_{i=1}^{k}q_{i}=\lambda \sum_{i=1}^{k}A^{H}\left(y-Ax_{i}\right)$$

The definition of CV-BI has been proved as mentioned above. The whole CV-BI can be summarized as follows:

(43) $$x^{k}=\arg\underset{x}{\min}H\left(x\right)+D_{J}^{p_{k-1}}\left(x,x_{k-1}\right)$$

(44) $$p_{k}=p_{k-1}-\nabla_{x^{*}}H\left(x^{k}\right)$$

 □

**Algorithm 1: CV-BI**
Initialization: *x*_0_ = 0 *p*_0_ = 0 *k* = 1 *λ*, 
  While “stopping criterion is not met” do     
$x^{k}=\arg\underset{x}{\min}H\left(x\right)+D_{J}^{p_{k-1}}\left(x,x_{k-1}\right)$;
$p_{k}=p_{k-1}-\nabla_{x^{*}}H\left(x^{k}\right)$;*K* = *k* + 1;End while

Review the entire process of proof and one can find that CV-BI possesses the ability to solve any kind of regularization term *J*(*x*) in Equation (18) if *J*(*x*) is a real convex function in complex variables. Furthermore, since each step of Algorithm 1 obeys the optimization rules in the complex domain instead of converting the objective function *Q_k_*(*x*) and variable *x* into the real domain, one can summarize that CV-BI preserve phase information for complex variables.

#### 3.3.3. Convergence Analysis

In this subsection, the convergence property of CV-BI is analyzed. To be specific, two monotonicity properties are proved with the help of the CV-BD.

**Proposition** **2.** *Under the above assumption, the sequence of H(x_k_) obtained from the CV-BI is monotonically nonincreasing, we get*

(45) $$H\left(x_{k}\right)\leq H\left(x_{k}\right)+D_{J}^{p_{k-1}}\left(x_{k},x_{k-1}\right)\leq H\left(x_{k-1}\right)$$

*Moreover, let x be such that J(x) < ∞, then we even have*

(46) $$D_{J}^{p_{k}}\left(x,x_{k}\right)+D_{J}^{p_{k-1}}\left(x_{k},x_{k-1}\right)+H\left(x_{k}\right)\leq H\left(x\right)+D_{J}^{p_{k-1}}\left(x,x_{k-1}\right)$$

**Proof.**  Recall the nonnegative property of the CV-BD and that *x_k_* is the minimizer of the convex function *Q_k_*(*x*), we obtain

(47) $$\begin{array}{ll} H\left(x_{k}\right) & \leq H\left(x_{k}\right)+J\left(x_{k}\right)-J\left(x_{k-1}\right)-2ℜ\left(\langle x_{k}-x_{k-1},p_{k-1}\rangle \right)\\ & =Q_{k}\left(x_{k}\right)\leq Q_{k}\left(x_{k-1}\right)=H\left(x_{k-1}\right) \end{array}$$

which implies Equation (45).

We can derive a formula motivated by the identity of the original BD [45]:

(48) $$\begin{array}{l} D_{J}^{p_{k}}\left(x,x_{k}\right)-D_{J}^{p_{k-1}}\left(x,x_{k-1}\right)+D_{J}^{p_{k}}\left(x_{k},x_{k-1}\right)\\ =J\left(x\right)-J\left(x_{k}\right)-2ℜ\left(\langle x-x_{k},p_{k}\rangle \right)\\ -J\left(x\right)+J\left(x_{k-1}\right)+2ℜ\left(\langle x-x_{k-1},p_{k-1}\rangle \right)\\ +J\left(x_{k}\right)-J\left(x_{k-1}\right)-2ℜ\left(\langle x_{k}-x_{k-1},p_{k-1}\rangle \right)\\ =2ℜ\left(\langle x-x_{k},p_{k-1}-p_{k}\rangle \right)\\ =2ℜ\left(\langle x-x_{k},q_{k}\rangle \right) \end{array}$$

Considering the definition of the CW subgradient and $q_{k}\in \partial_{x^{*}}H\left(x_{k}\right)$, we yield

(49) $$\begin{array}{ll} D_{J}^{p_{k}}\left(x,x_{k}\right)-D_{J}^{p_{k-1}}\left(x,x_{k-1}\right)+D_{J}^{p_{k}}\left(x_{k},x_{k-1}\right) & =2ℜ\left(\langle x-x_{k},q_{k}\rangle \right)\\ & \leq H\left(x\right)-H\left(x_{k}\right) \end{array}$$

which is equivalent to Equation (46). □

**Proposition** **3.**  *Under the same assumption as Proposition 2, let* $\tilde{x}$ *be a minimizer of H(x) with* $J(\tilde{x})<\infty$ *, then we have*

(50) $$D_{J}^{p_{k}}\left(\tilde{x},x_{k}\right)\leq D_{J}^{p_{k-1}}\left(\tilde{x},x_{k-1}\right)$$

**Proof.**  Recall the nonnegative property of the CV-BD and that $\tilde{x}$ is a minimizer of *H*(*x*), we get an inequality

(51) $$D_{J}^{p_{k}}\left(\tilde{x},x_{k}\right)\leq D_{J}^{p_{k}}\left(\tilde{x},x_{k}\right)+D_{J}^{p_{k-1}}\left(x_{k},x_{k-1}\right)+H\left(x_{k}\right)-H\left(\tilde{x}\right)$$

According to Equations (49), (51) can be derived as

(52) $$D_{J}^{p_{k}}\left(\tilde{x},x_{k}\right)\leq D_{J}^{p_{k}}\left(\tilde{x},x_{k}\right)+D_{J}^{p_{k-1}}\left(x_{k},x_{k-1}\right)+H\left(x_{k}\right)-H\left(\tilde{x}\right)\leq D_{J}^{p_{k-1}}\left(\tilde{x},x_{k-1}\right)$$

which proves Equation (50). The results of Equations (45) and (50) conclude a general convergence conclusion for CV-BI. More details about convergence can be found in [29]. □

### 3.4. CV-SBM

For various kinds of regularization terms corresponding to Equation (43), CV-BI still has to employ other algorithms as BI does, which makes the solution process complicated and computationally expensive. Inspired by SBM, we separate the original variable and present CV-SBM to settle the convex inverse problems with the simplified solutions.

A constrained optimization problem in complex variables

(53) $$\underset{x,d\in {\mathbb{C}}^{n}}{\min}J\left(d\right)+H\left(x\right)\;s.t.\;d=\Phi x$$

can be transformed into an unconstrained one

(54) $$\begin{array}{l} \underset{x,d\in {\mathbb{C}}^{n}}{\min}F\left(x,d\right)+\mu {\left\|d-\Phi x\right\|}_{2}^{2}\\ F\left(x,d\right)=J\left(d\right)+H\left(x\right) \end{array}$$

Evidently, *F*(*x*,*d*) is convex in *x* and *d*. Thus, by applying CV-BI to Equation (54) in each variable, we can derive that

(55) $$\begin{array}{ll} \left(x^{k},d^{k}\right) & =\arg\underset{x,d}{\min}D_{F}^{p_{k-1}}\left(x,x^{k-1},d,d^{k-1}\right)+\mu {\left\|d-\Phi x\right\|}_{2}^{2}\\ & =\arg\underset{x,d}{\min}F\left(x,d\right)-F\left(x^{k-1},d^{k-1}\right)\\ & -2ℜ\left(\langle x-x^{k-1},p_{x}^{k-1}\rangle \right)-2ℜ\left(\langle d-d^{k-1},p_{d}^{k-1}\rangle \right)+\mu {\left\|d-\Phi x\right\|}_{2}^{2} \end{array}$$

(56) $$p_{x}^{k}=p_{x}^{k-1}-\mu \Phi^{H}\left(\Phi x^{k}-d^{k}\right)$$

(57) $$p_{d}^{k}=p_{d}^{k-1}-\mu \left(d^{k}-\Phi x^{k}\right)$$

To simplify the above iteration step Equation (55), we assume $b^{k}=p_{d}^{k}/\mu$ and get

(58) $$\begin{array}{l} \;-2ℜ\left(\langle x-x^{k-1},p_{x}^{k-1}\rangle \right)-2ℜ\left(\langle d-d^{k-1},p_{d}^{k-1}\rangle \right)+\mu {\left\|d-\Phi x\right\|}_{2}^{2}\\ =2\mu ℜ\left(\langle x-x^{k-1},\Phi^{H}b^{k-1}\rangle \right)-2\mu ℜ\left(\langle d-d^{k-1},b^{k-1}\rangle \right)+\mu {\left\|d-\Phi x\right\|}_{2}^{2}\\ =2\mu ℜ\left(\langle \Phi x-\Phi x^{k-1},b^{k-1}\rangle \right)-2\mu ℜ\left(\langle d-d^{k-1},b^{k-1}\rangle \right)+\mu {\left\|d-\Phi x\right\|}_{2}^{2}\\ =\mu \left[2ℜ\left(\langle \Phi x-d,b^{k-1}\rangle \right)-2ℜ\left(\langle \Phi x^{k-1}-d^{k-1},b^{k-1}\rangle \right)+{\left\|d-\Phi x\right\|}_{2}^{2}\right]\\ =\mu \left[2ℜ\left(\langle \Phi x-d,b^{k-1}\rangle \right)+{\left\|d-\Phi x\right\|}_{2}^{2}\right]+C_{1}\\ =\mu \left[\langle \Phi x-d,b^{k-1}\rangle +{\langle \Phi x-d,b^{k-1}\rangle }^{*}+{\left\|d-\Phi x\right\|}_{2}^{2}\right]+C_{1}\\ =\mu \left[{\left(b^{k-1}\right)}^{H}\left(\Phi x-d\right)+{\left(\Phi x-d\right)}^{H}b^{k-1}+{\left\|d-\Phi x\right\|}_{2}^{2}\right]+C_{1}\\ =\mu {\left\|d-\Phi x-b^{k-1}\right\|}_{2}^{2}+C_{1} \end{array}$$

where *C*_1_ is a constant. Substituting Equation (58) in Equations (55)–(57) yields

(59) $$\left(x^{k},d^{k}\right)=\arg\underset{x,d}{\min}F\left(x,d\right)+\mu {\left\|d-\Phi x-b^{k-1}\right\|}_{2}^{2}$$

(60) $$b^{k}=b^{k-1}-\left(d^{k}-\Phi x^{k}\right)$$

One can resolve Equation (59) by alternating minimization scheme with respect to *x* and *d*

(61) $$x^{k}=\arg\underset{x}{\min}H\left(x\right)+\frac{\mu }{2}{\left\|d^{k-1}-\Phi x-b^{k-1}\right\|}_{2}^{2}$$

(62) $$d^{k}=\arg\underset{d}{\min}J\left(d\right)+\frac{\mu }{2}{\left\|d-\Phi x^{k}-b^{k-1}\right\|}_{2}^{2}$$

The above two subproblems can be worked out easily. Considering the property of CV-BI, it can be inferred that CV-SBM is also universal for convex *J*(*x*) in any convex optimization task.

The overall CV-SBM is shown as Algorithm 2


**Algorithm 2: CV-SBM**
Initialization: *x*_0_ = 0, *d*_0_ = 0, *p*_0_ = 0, *λ*, *μ*, *k* = 1  While “stopping criterion is not met” do    $x^{k}=\arg\underset{x}{\min}H\left(x\right)+\frac{\mu }{2}{\left\|d^{k-1}-\Phi x-b^{k-1}\right\|}_{2}^{2}$;$d^{k}=\arg\underset{d}{\min}{\left\|d\right\|}_{1}+\frac{\mu }{2}{\left\|d-\Phi x^{k}-b^{k-1}\right\|}_{2}^{2}$;$b^{k}=b^{k-1}-\left(d^{k}-\Phi x^{k}\right)$;*k* =*k*+1;End while

Assuming Φ = I, then we can work out Equation (18) through CV-SBM by three steps [35]:

Step1: Clear up the *x* subproblem

(63) $$x^{k}=\arg\underset{x\in {\mathbb{C}}^{n}}{\min}\lambda {\left\|y-Ax\right\|}_{2}^{2}+\mu {\left\|d^{k-1}-x-b^{k-1}\right\|}_{2}^{2}$$

Considering the *l*_2_-norm is differentiable, Equation (63) can be tackled by taking the CW-gradient of *x* equal to zero, and yield

(64) $$x^{k}={\left(\lambda A^{T}A+\mu I\right)}^{-1}\left(\lambda A^{T}y+\mu d^{k-1}-\mu b^{k-1}\right)$$

Step2: Find a solution to the *d* subproblem

(65) $$d^{k}=\arg\underset{d\in {\mathbb{C}}^{n}}{\min}{\left\|d\right\|}_{1}+\frac{\mu }{2}{\left\|d-x^{k}-b^{k-1}\right\|}_{2}^{2}$$

This subproblem can be dealt with by a shrinkage operator

(66) $$d^{k}=shrink\left(x^{k}+b^{k-1},1/\mu \right)$$

(67) shrink(γ,η)=sign(γ)max(|γ|−η,0)

Step3: Update *b*

(68) $$b^{k}=b^{k-1}-\left(d^{k}-x^{k}\right)$$

CV-SBM for *l*_1_-norm problem can be presented as Algorithm 3


**Algorithm 3: CV-SBM for l1-norm problem**
Initialization: *x*_0_=0, *d*_0_=0, *p*_0_=0, *λ*, *μ*, *k*=1  While “stopping criterion is not met” do    $x^{k}={\left(\lambda A^{T}A+\mu I\right)}^{-1}\left(\lambda A^{T}y+\mu d^{k-1}-\mu b^{k-1}\right)$;    $d^{k}=shrink\left(x^{k}+b^{k-1},1/\mu \right)$;    $b^{k}=b^{k-1}-\left(d^{k}-x^{k}\right)$;    *k* = *k*+1;    End while

## 4. Numerical Experiments

This section presents the performance of the proposed CV-SBM by conducting a wide range of experiments solving *l*_1_-norm problems in the complex domain. We apply the proposed method to recover a complex-valued random sparse signal *x* from the noisy measurements *y* generated by *y* = *Ax* + *ε*, where $x\in {\mathbb{C}}^{n}$, $y\in {\mathbb{C}}^{m}$, $A\in {\mathbb{C}}^{m\times n}$, $\varepsilon \in {\mathbb{C}}^{m}$. The sparse signal *x* consists of *L* nonzero elements and the amplitudes of both *x*’s and *A*’s real part and imaginary part obey Gaussian Distribution *N* (0,1). The noise vector *ε* is assumed to be i.i.d zero-mean complex Gaussian noise. The contrastive means for the proposed scheme in the following subsections are as follows: classical OMP [46], CAMP, M-lasso, and the original SBM converting technique [47]. Noted that in the following, the original SBM is called RV-SBM. In addition, Section 4.1.2 presents the performance of the proposed method conducted in ISAR imaging.

The stopping criterion for all algorithms is given as follow

(69) $$\frac{{\left\|x^{k}-x^{k-1}\right\|}_{2}^{2}}{{\left\|x^{k-1}\right\|}_{2}^{2}}\leq tol$$

or

(70) $$\;k=k_{\max}$$

where *tol* = 2e^−4^ denotes the tolerance and *k*_max_ = 2000 is the maximum iteration times. All the experiments are carried out in MATLAB 2016b on the PC with Intel I7 7700K @4.2 GHz with 32 GB memory.

### 4.1. An Illustrative Example

#### 4.1.1. Complex-Valued Random Sparse Signal Recovery

In this subsection, an illustrative example is devised to demonstrate the effectiveness of the proposed method in comparison with OMP, CAMP, M-lasso, and RV-SBM. We consider that the signal and measurement dimension is *n* = 256 and *m* = 128, respectively. Moreover, the sparsity level of *x* is fixed *L* = 32 and the Signal to Noise Ratio (SNR) is set to 15 dB.

**Figure 1:**
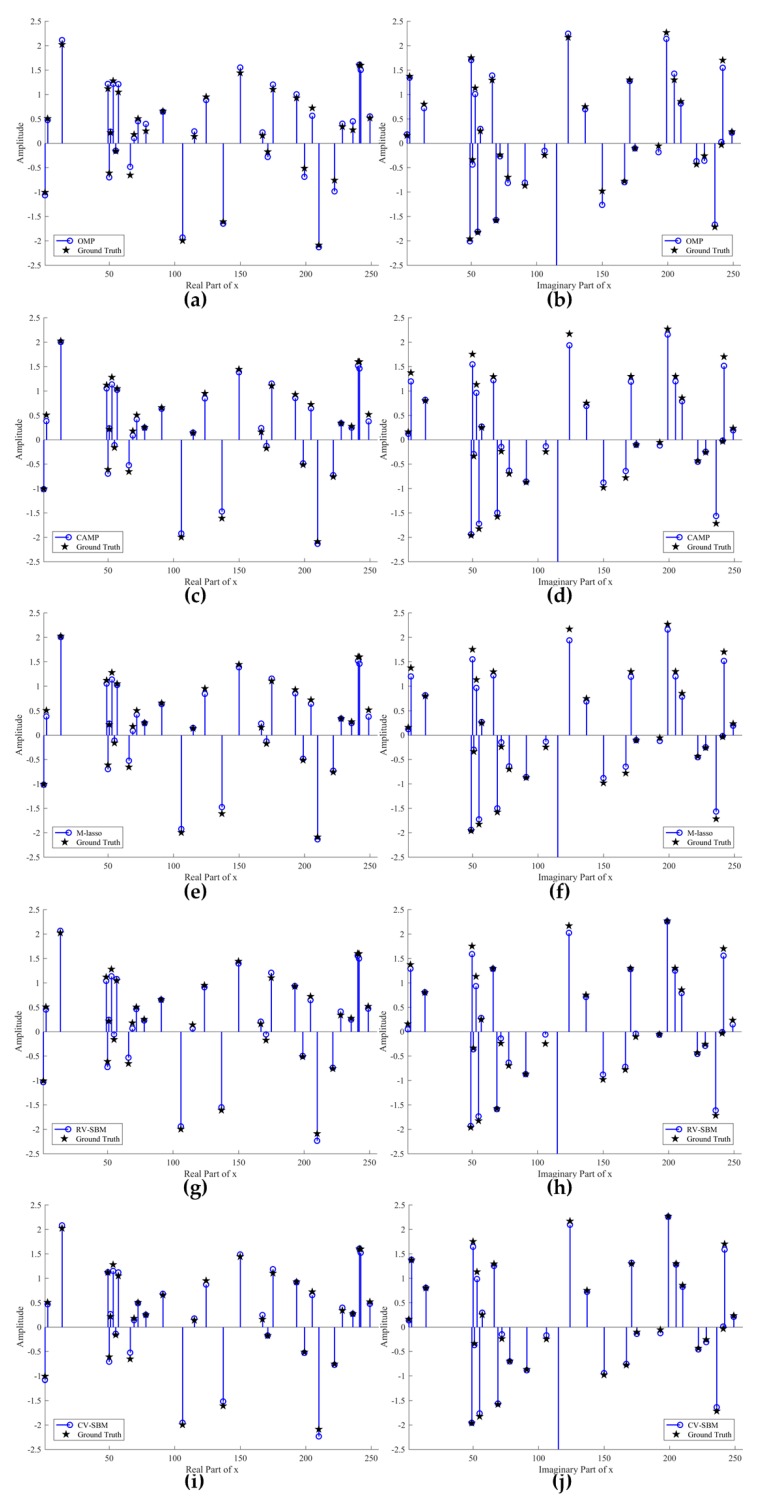
Comparison of the real and imaginary parts of the reconstruction results by OMP, CAMP, M-lasso, RV-SBM, and the proposed CV-SBM: (**a**) recovery performance for the real part of *x* by OMP; (**b**) recovery performance for the imaginary part of *x* by OMP; (**c**) recovery performance for the real part of *x* by CAMP; (**d**) recovery performance for the imaginary part of *x* by CAMP; (**e**) recovery performance for the real part of *x* by M-lasso; (**f**) recovery performance for the imaginary part of *x* by M-lasso; (**g**) recovery performance for the real part of *x* by RV-SBM; (**h**) recovery performance for the imaginary part of *x* by RV-SBM; (**i**) recovery performance for the real part of *x* by CV-SBM; (**j**) recovery performance for the imaginary part of *x* by CV-SBM.

Figure 1 shows the contrast to the real and imaginary part of reconstruction signal among the contrastive means and the proposed CV-SBM. The blue circle lines represent the recovered signal and the black stars denote the ground truth. Note that zero-valued points of *x* remain hidden to emphasize the nonzero ones in Figure 1. As shown in Figure 1a,b, there are five and nine accurately reconstructed points (circle and star coincide) in real and imaginary part achieved by OMP, respectively. Unsurprisingly, plenty of points mismatch far away from their position, especially the 150th point in the imaginary domain. Figure 1c,d exhibits the reconstruction result of CAMP, which yields nine well-recovered points in both the real and imaginary parts. However, there also exist outliers, but less than OMP’s. In Figure 1e,f, eight and nine points are accurately recovered in the real and imaginary domains by M-lasso, respectively. It can be seen that CAMP and M-lasso behave almost the same, better than OMP. Figure 1g,h give the recovery results for the original RV-SBM whose real part shows eight well-reconstructed points and whose imaginary part demonstrates 11 points. The proposed technique yields 10 and 15 accurately recovered points, shown in Figure 1i,j, the most among the algorithms. Comparison of the number of accurately reconstructed points is presented in Table 1. In addition, the furthest outlier given by CV-SBM is at the same length as RV-SBM’s but far less than the others’. This proves the effectiveness of CV-SBM for complex sparse signal recovery.

#### 4.1.2. ISAR Imaging with Real Data

**Figure 2:**
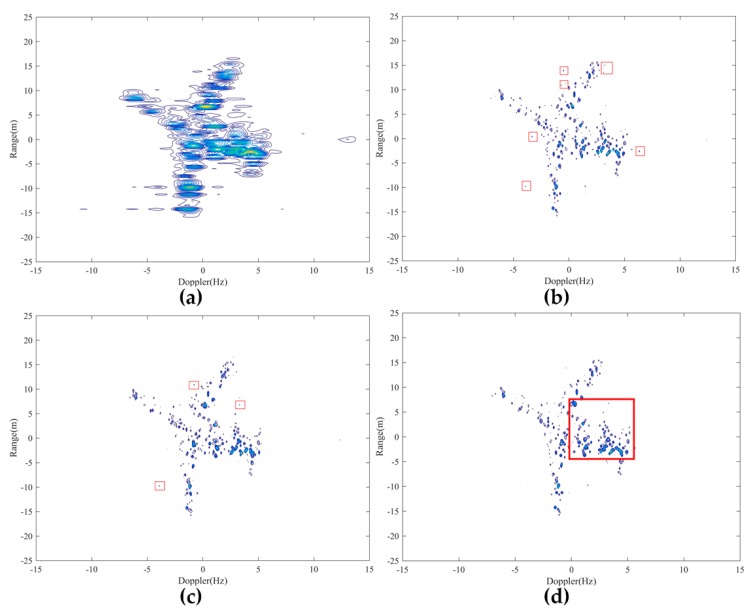
Comparison of ISAR imaging by RD [48], RV-SBM, and the proposed CV-SBM: (**a**) imaging result by RD; (**b**) imaging result by [48]; (**c**) imaging result by RV-SBM; (**d**) imaging result by CV-SBM.

In this subsection, CV-SBM is applied in ISAR imaging with real data of the Yak-42 plane to demonstrate its superiority, comparing with RV-SBM, the range-Doppler (RD) algorithm, and the CS recovery method [48]. Detailed descriptions of targets and data are provided in [49]. Main radar parameters are listed as follows: The signal bandwidth is 400 MHz with carrier frequency 10 GHz, corresponding to a range resolution of 0.375 m. The pulse repetition frequency is 100 Hz, i.e., 64 pulses within dwell time [−0.32, 0.32] (s) are used in this experiment. Motion compensated data are utilized by the aforementioned four algorithms, shown in Figure 2a–d.

Figure 2a exhibits the result of the RD algorithm, in which low-quality focal and high side-lobes occur. In Figure 2b, many strong scatters are extracted by [48]. However, there still exist several strong outliers marked in red boxes. Figure 2c indicates the target’s geometry. Besides, the number of outliers recovered by RV-SBM is less than [48]. In Figure 2d, the target’s geometry is clear and scatters, marked in red box, extracted by CV-SBM are stronger than ones in the same area by [48] and RV-SBM. Furthermore, most of the outliers shown in Figure 2b,c are suppressed greatly by the proposed CV-SBM. This proves the effectiveness of CV-SBM in real data processing of ISAR imaging.

### 4.2. Robustness Against Measurement Noise

**Figure 3:**
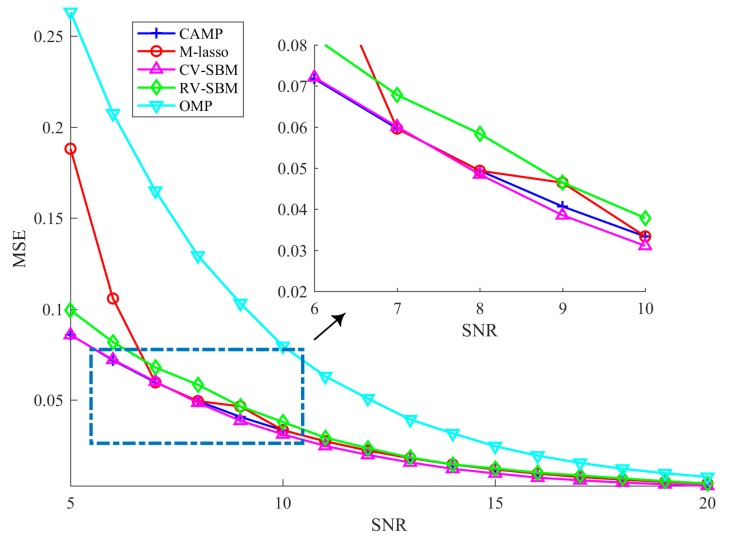
Average MSE in different measurement noise levels.

In this subsection, we test the robustness of the proposed technique against the measurement noise. The experimental parameters are set as follows: SNR varies from 5 dB to 20 dB and other parameters are fixed the same as in the previous subsection. For each SNR, we average the MSE of 100 independent trials as the experimental result, as shown in Figure 3.

As the SNR increases, the MSE of the proposed scheme declines, which implies CV-SBM is robust to the noise. Before the SNR reaches 7 dB, the MSEs of CAMP and CV-SBM are almost the same, but when SNR goes beyond 7 dB, the MSE of CV-SBM surpasses CAMP’s and becomes the lowest among all the algorithms. Both OMP’s and RV-SBM’s MSE numerically exceed CV-SBM’s. In addition, CV-SBM behaves better than M-lasso, except at the point when the SNR is equal to 7 dB, at which they are approximately the same. This demonstrates that the proposed algorithm has better robustness against the measurement noise among the methods.

### 4.3. Robustness Against Measurement Noise

**Figure 4:**
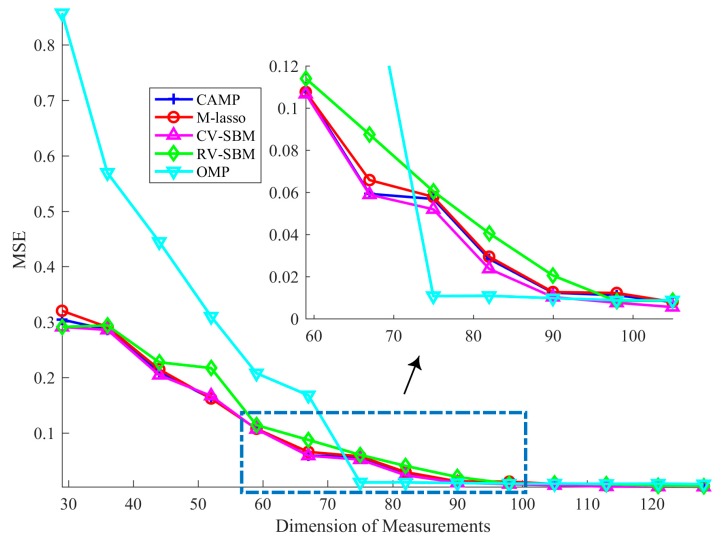
Average MSE in different measurements dimensions.

In this subsection, how the dimension of measurements influences the recovery result is presented. We set *n* = 256, SNR = 15 dB, *L* = 32, and *m* varies from 29 to 128. As in the previous subsection, we measure the average MSE over 100 independent trials, as shown in Figure 4. It can be seen that as the dimension of the measurements rises, the MSE of CV-SBM decreases, which means that the larger the dimension of the measurements, the better the recovery performance of the proposed method.

In Figure 4, the MSE of CV-SBM is lower than RV-SBM’s and M-lasso’s. Except when the measurement dimension is equal to 52 and 59, the performance of the proposed method is better than CAMP’s. Before the dimension reaches 75, the MSE of OMP is far worse than CV-SBM’s. However, when the dimension exceeds 75, the MSE of OMP reaches to 0.01 suddenly but reduces slowly as it improves, becoming equal to CV-SBM’s at 90. When the dimension is larger than 90, the MSE of CV-SBM continues to decline and becomes the lowest among all the algorithms.

### 4.4. Time Cost Assessment

**Figure 5:**
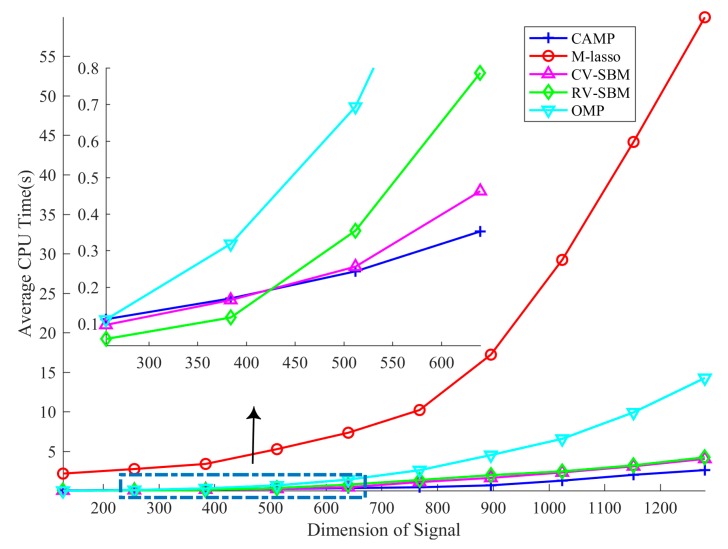
Average CPU time cost in different signal dimensions.

In this subsection, the computational cost of the proposed method is measured with increasing dimension of the signal. To this end, we vary *n* from 128 to 1024 and fix SNR = 20 dB, *m* = 0.5*n*, *L* = 0.125*n*. For each *n*, we conduct 20 independent trials and average the CPU time cost as the result, as shown in Figure 5.

The result shows that CV-SBM takes less CPU time than OMP and M-lasso in all test dimensions. Before the dimension reaches 512, RV-SBM requires the least time. However, when the dimension is more than 512, CV-SBM requires less CPU time than RV-SBM. This is because the complex-to-real transformation utilized in RV-SBM expands the dimension of the sensing matrix *A* to 2*m* × 2*n*, which leads to an inverse matrix with 2*n* × 2*n* elements and takes more memory and time within the resolving process, while CV-SBM only needs to calculate a complex inverse matrix with *n* × *n* elements. Nevertheless, CV-SBM takes a little more time than CAMP in large signal scale situations thanks to CAMP’s specific design for *l*_1_-norm problems, whereas CV-SBM contains an inverse operator. However, CV-SBM still has great potential to exceed CAMP considering that the gap between CAMP and CV-SBM is not large.

### 4.5. Performance Comparison with RV-SBM

The tests mentioned above have shown that the proposed CV-SBM presents remarkable performance compared with RV-SBM in the same experimental environment. Thus, in this subsection, we focus on the convergence, time cost, and performance of CV-SBM and RV-SBM by implementing experiments with various parameters. In the following experiments, two main parameters for CV-SBM and RV-SBM are set to *λ* = 0.005 and *μ* = 120 and the stopping criterion (the tolerance *tol* and *k*_max_) varies. Furthermore, other experimental parameters are as follow: *L* = 0.125*n*, *m* = 0.5*n* and SNR varies from 10 dB, 15 dB, and 20 dB. For each stopping criterion and SNR, 20 independent trials were carried out and the average MSE, CPU time cost, and iteration time are selected as the result. The average MSE and CPU time cost of the proposed method are also presented if CV-SBM presents better performance.

In the first test, we examine CV-SBM and RV-SBM in small scale *n* = 256 and fix *tol* = 2e^−4^, *k*_max_ = 2000, as shown in Table 2. It can be seen that in each SNR situation, in the vast majority of trials, RV-SBM achieves the stopping criterion Equation (69) and requires less CPU time and fewer iterations, while the MSE of CV-SBM is always superior. This implies that RV-SBM possesses more rapid convergence, but this property also leads to a severe performance loss. Besides, the convergence speed of CV-SBM is about five times slower than that of RV-SBM, but this provides CV-SBM more iterations to attain better performance. It should be pointed out that the average CPU time of RV-SBM is not five times as fast as CV-SBM because a minority of trials of RV-SBM still consume 2000 iterations.

To inspect the performance regardless of the convergence condition, we reduce the tolerance to *tol* = 2e^−5^ and keep the other parameters consistent with the previous experiment (Table 3). For any situation in Table 3, both RV-SBM and CV-SBM stop by reaching the maximum iterations. As we can see, the MSE gap between CV-SBM and RV-SBM is extremely narrow, but still exists. This illustrates that CV-SBM achieves better performance due to employing the phase information. Furthermore, CV-SBM still requires a little more time than RV-SBM, as in the first experiment. 

At last, large scale *n* = 512 is taken into consideration (Table 4). The tolerance *tol* and the maximum iteration times *k*_max_ are set the same as in Table 3. CV-SBM achieves superior performance in terms of both MSE and time cost in comparison with RV-SBM. Specifically, CV-SBM yields 18.20%, 17.58%, and 26.67% lower MSE and requires 28.75%, 25.59%, and 23.64% less CPU time than RV-SBM in all kinds of SNR cases, respectively. This reveals that the proposed CV-SBM is extremely applicable in large-scale complex-valued sparse signal recovery.

## 5. Conclusions

In this paper, a new CS recovery algorithm named CV-SBM is presented, which generalizes the widely employed SBM into the complex domain. CV-SBM induces a theoretical support to directly reconstruct the sparse signal in complex-valued variables, instead of converting them into real ones. We apply the proposed CV-SBM to a *l*_1_-norm problem to recover a complex-valued sparse signal. Experimental results demonstrate the superiority of CV-SBM over other existing CS reconstruction methods, especially RV-SBM, in both recovery accuracy and time cost for large-scale cases.

A significant goal for future work lies in applying CV-SBM to more complicated regularization problems, since CV-BI and CV-SBM are theoretically able to deal with any convex optimization problem.

## Figures and Tables

**Table 1:** Comparison of recovery performance by OMP, CAMP, M-lasso, RV-SBM, and the proposed CV-SBM.

	Number of Well-Recovered Points	
	Real Part of *x*	Imaginary Part of *x*
**OMP**	5	9
**CAMP**	9	9
**M-lasso**	8	9
**RV-SBM**	8	11
**CV-SBM**	10	15

**Table 2:** Comparison of CV-SBM and RV-SBM when *tol* = 2e^−4^, *k*_max_ = 2000, and *n* = 256.

SNR (dB)	Average MSE			Average CPU Time (s)			Average Iterations	
	CV-SBM	RV-SBM	Promotion	CV-SBM	RV-SBM	Promotion	CV-SBM	RV-SBM
10 dB	0.0426	0.0592	28.04%	0.0892	0.0258	N/A	2000.0	728.4
15 dB	0.0125	0.0523	76.10%	0.0885	0.0167	N/A	1991.3	387.3
20 dB	0.0031	0.0484	93.60%	0.0766	0.0167	N/A	1740.9	375.3

**Table 3:** Comparison of CV-SBM and RV-SBM when *tol* = 2e^−5^, *k*_max_ = 2000, and *n* = 256.

SNR (dB)	Average MSE			Average CPU Time (s)			Average Iterations	
	CV-SBM	RV-SBM	Promotion	CV-SBM	RV-SBM	Promotion	CV-SBM	RV-SBM
10 dB	0.0426	0.0489	12.88%	0.0892	0.0537	N/A	2000	2000
15 dB	0.0125	0.0146	14.38%	0.0874	0.0519	N/A	2000	2000
20 dB	0.0029	0.0042	30.95%	0.0865	0.0517	N/A	2000	2000

**Table 4:** Comparison of CV-SBM and RV-SBM when *tol* = 2e^−4^, *k*_max_ = 2000 and *n* = 512.

SNR(dB)	Average MSE			Average CPU Time(s)			Average Iterations	
	CV-SBM	RV-SBM	Promotion	CV-SBM	RV-SBM	Promotion	CV-SBM	RV-SBM
10dB	0.0445	0.0544	18.20%	0.2706	0.3798	28.75%	2000	2000
15dB	0.0136	0.0165	17.58%	0.2608	0.3505	25.59%	2000	2000
20dB	0.0033	0.0045	26.67%	0.2633	0.3448	23.64%	2000	2000
